# The *Tout-Monde* of disaster studies

**DOI:** 10.4102/jamba.v15i1.1385

**Published:** 2023-02-22

**Authors:** JC Gaillard

**Affiliations:** 1 Te Kura Mātai Taiao, Waipapa Taumata Rau, Aotearoa

**Keywords:** disaster studies, postcolonial studies, pluralism, relation, Edouard Glissant

## Abstract

This essay expands the postcolonial agenda for future disaster studies that we suggested in the conclusion of the book *The Invention of Disaster*. It provides some refined perspectives on how to capture the diversity and complexity of the world that we draw from the philosophy of Martinican poet and novelist Edouard Glissant. Glissant’s philosophy of creolisation and relation offers critical pathways towards pluralistic approaches to understanding what we call disaster in a world that is marked by hybridity and relationships rather than essentialism and nativism. A *Tout-Monde*, in Glissant’s terms, that is the combined additions of different and hybrid interpretations of disaster.

**Contribution:**

Exploring the *Tout-Monde* of disaster studies will constitute a radical and forward-looking postcolonial agenda; radical in the sense that it will challenge many of our scholarly assumptions, popular discourses as well as common-sense policies and practices.

‘Comment être soi sans se fermer à l’autre, et comment s’ouvrir à l’autre sans se perdre soi-même?’^1^

Glissant (1995:20)

## On the hegemony of Western discourses on disaster

We have recently argued that our dominant understandings of disaster are largely informed by Western, that is, Eurocentric, perspective on the world (Gaillard 2021). The very concept of disaster is indeed an invention of the West^2^ that reflects the injunction of the project of modernity, as envisioned in Europe’s 18th century, to free people from the threats of nature so that they can flourish in life. As a result, contemporary discourses on disaster are firmly grounded in the ontological assumption that such disasters sit at the interface between nature and society, or, in the lingua of disaster scholarship, between hazard and vulnerability. A perfect example of Escobar’s (2018:4) ‘hegemony of modernity’s one-world ontology’.

Theoretical and epistemological debates on whether disasters are the consequence of nature/hazards or society/vulnerability are all bounded by this ontological assumption and only constitute a pendulum shift within a binary understanding of the world; one that is Eurocentric in nature. As such, all existing paradigms that have been put forward to understand people’s experiences of what we call disaster continue to perpetuate the hegemony of Western scholarship and mirror Hegel’s dialectical quest for reason (Gaillard 2021). As if there could be one single and universal truth when it comes to disaster, which is the imperial truth of the West according to Eboussi-Boulaga (1977).^3^ In this context, arguing that disasters are social constructs appear an epistemological non-sense. How, indeed, could we rely on one single set of ethnocentric and universal theories, concepts and methodologies, although diverse, to understand the experiences of millions of people across very different cultures and societies around the world?

Although antithetical and non-sensical, the hegemony of Western discourses on disaster has nonetheless sustained standardised and universal policies and actions for disaster risk reduction all over the world. This prolongs the imperialist agenda of the West, in dealing with what we call disasters and beyond, as brilliantly exposed by Bankoff (2001, 2019). Therefore, the shift observed over the past 30 years from technocratic, military-inspired and science or technology-informed policies and actions to so-called community-based and participatory processes often (not always) constitute no more than the reproduction of the pendulum swing evident within disaster studies. Consequently, disaster risk reduction initiatives, in all their diversity, continue to promote Western discourses geared to meeting the injunction of the project of modernity, that is, to free people from the dangers of nature.

In *The Invention of Disaster*, our intention was to unpack the ontological, epistemological and political processes that have made possible and eventually sustained this hegemony of Western discourses on disaster; an endeavour of deconstruction in Derrida’s (2004) words. Only in the book’s conclusion, we outlined some leads towards more grounded, genuine and, henceforth, meaningful disaster studies in the future. It is our intention in the present essay to expand this postcolonial agenda and provide some refined perspectives on how to capture the diversity and complexity of the world, especially when it comes to understanding what we call disaster, if ever the said concept makes sense in places beyond the West.

We particularly draw on the philosophy of Martinican poet and novelist Edouard Glissant. Glissant’s philosophy of creolisation and relation allows to expand the rationale for the approach informed by postcolonial studies and based on dialogue that we outlined in the conclusion of *The Invention of Disaster*. It further offers critical pathways towards pluralistic approaches to understanding what we call disaster in a world that is marked by hybridity and relationships rather than essentialism and nativism.

## For oneself and by oneself

The concept of disaster is an invention of the West because it does not refer to any transcendental signifier, in Derrida’s (1967) sense. Rather, it relies on the deferred understanding of other concepts that point to different things: Derrida’s *différance* [deferring] and *difference* [difference]. Indeed, understanding disaster on Western terms requires that one understand what a hazard, that is, a potentially threatening phenomenon and vulnerability, that is, the susceptibility to suffer, are. Understanding the latter concepts further necessitates that one understands what danger and harm are, etc. The problem is that all these concepts rely on subjective interpretations of life. Indeed, what is acceptable for one individual or one group of people is possibly not for others, whether these share common social and cultural traits or not.

Nonetheless, contemporary discourses on disaster, in their diversity, rely on normative interpretations of life as per the expectations of 18th-century Europe’s project of modernity, that is, people’s ability to flourish should not be impeded by the hazards of nature. As a result, disasters, whether understood from the so-called hazard or vulnerability paradigms, are processes and situations that prevent people to ‘grow’ and ‘enjoy’ everyday life. In this perspective, normative thresholds are drawn after Western standards between what is acceptable suffering, damage and harm, the *dis*- in the -aster, in a process similar to the drawing of an artificial line for *dis*-ease, *dis*-ability and mental *dis*-order (Canguilhem 1966). This drawing of a line between what is acceptable and not is inherently subjective, as, for example, in the lack of consensus in the criteria of the multiple databases and indexes used in the field of disaster studies, including those that inform praxis (Gaillard 2021).

Revisiting disaster studies and our understanding of disaster from a postcolonial perspective, that is, beyond such universal assumptions about life (Spivak 1993), requires to reconsider normative definitions and expectations about what is acceptable harm and suffering. It thus requires reclaiming subjectivity or, in Senghor’s (1971) and Eboussi-Boulaga’s (1977) terms, that one be able to think for oneself and by oneself; that one does not have to think anymore using others’ worldviews or senses to serve others’ interests, nor to think anymore for oneself using others’ worldviews or senses; two perspectives similarly skewed towards Western ontologies and epistemologies (Salazar 1991).

This is a liberation agenda. A liberation of the subject from the imperialist subjugation to universal Western world views (Foucault 1978, 1997). A liberation that is open and forward looking rather than dialectical and driven by the past. It is in this perspective that our agenda is postcolonial rather than decolonial. If we recognise that the emancipating nature of the decolonial agenda could be an important first step and, henceforth, cannot be neglected for its purifying effect in places still subjected to colonial or neocolonial power (Tuhiwai-Smith 2012), we nonetheless suggest that we should move beyond solely pushing back against the past in a dialectical perspective that is inherently inhibited by the colonial dialectic and its skewed and hierarchical understanding of difference (Bhabha 1994; Memmi 1957). As such, our intention differs from Wiredu’s (1980) decolonial agenda to understanding the world in the postcolony, that is, to start with Western concepts and explore their relevance beyond the West. We rather suggest starting from local understandings of the world and explore opportunities forward. It is about re-imagining the present and envisioning the future, not only rejecting the past.

Moving towards this postcolonial agenda requires for local and grounded scholars to move away from the hegemony of Western ontologies and epistemologies that have underpinned disaster studies so far. It necessitates to ground concepts and methodologies in local people’s understanding of the world, whether indigenous or hybrid as in many locations of the world nowadays. Such a pluralist approach to interpreting disaster, if ever the concept locally makes sense, is at the core of the postcolonial agenda that we proposed in the conclusion of *The Invention of Disaster*. It innately requires recognition and acceptance of otherness^4^ and difference, two essential dimensions of Glissant’s philosophy.

## Towards plural understandings of what we call disaster

In *The Invention of Disaster*, our goal was to trace the genealogy of the current *métarecits* of disaster and how they have underpinned broader discourses that have shaped the praxis of disaster risk reduction. We further argued that the seeming differences between academic paradigms and approaches to policies and actions are in fact all bound by the ontological limitations of the nature/hazard-society/vulnerability binary and hence stuck within a Western understanding of the world.

Our contention, in this essay, is that we need to go beyond this ontological assumption and open ourselves to other narratives of disaster. There may indeed be multiple diverse interpretations of what we call disaster and these may reflect different understandings of the world. These other interpretations may or may not relate to the ontological assumption that disasters sit at the interface between nature and society. Recognising such diversity in our interpretations of what we call disaster requires to reconsider our understanding of the world at large and of our more immediate environment in particular. It is inherently an ontological and epistemological matter. Therefore, there cannot be any *métarécits* or grand narratives of disaster, neither can there be any models nor standardised methodologies to study them.

Our agenda is therefore about ‘ontological pluralism’, in Latour’s (2012:150) sense, that is, there can be multiple modes of existence, multiple ways of understanding and making sense of the world, including of natural phenomena, harm and suffering. This, in no way, challenges the relevance and strength of Western interpretations of disaster within their cradle and among people who claim European descendancy. Nor do we meant to dismiss or overlook people’s suffering in facing natural phenomena that may bring harm. Rather, we contend that there cannot be one single way of experiencing such harm and suffering when confronted with natural and other processes, human relationships and broader challenges in life. It is about challenging the hegemony of a ‘one-world ontology’ (Escobar 2018).

The concept of mode of existence or mode of being is here crucial because, as we reminded earlier, there are no transcendental signifiers for a disaster, which inherently reflects subjective expectations about life, that is, how we interpret and experience harm and suffering. In his landmark exploration of the normal and pathological in life and medicine, Canguilhem (1966) once argued that:

[*L*]a frontière entre le normal et le pathologique est imprécise pour des individus multiples considérés simultanément, mais elle est parfaitement précise pour un seul et même individu considéré successivement. Ce qui est normal, pour être normatif dans des conditions données, peut devenir pathologique dans une autre situation, s’il se maintient identique à soi. De cette transformation c’est l’individu qui est juge parce que c’est lui qui en pâtit, au moment même où il se sent inférieur aux tâches que la situation nouvelle lui propose.^5^ (p. 156)

The same applies to disaster and how people interpret harm and suffering in dealing with challenges in life, whether associated with natural phenomena or any other possible threats. Such interpretation and experience of harm and suffering are set against the normative expectations of our diverse societies, which, in turn, are affected by cultural values, religious beliefs, kinship, etc.

Thus, there cannot be any absolute and universal truth about disaster, especially if it is to be informed by one single form of reason as carved by Western science and the project of modernity of Europe’s 18th century. Rather, we argue that there are multiple truths, all rational rather than subjective because grounded in local understandings of the world. All grounded and underpinned by different *épistémès*, which, in Foucault’s (1966) terms, support the formation of knowledge and discourses that validate truth. A case also made by the proponents of pluriversality in Latin America and beyond (Escobar 2018; Mignolo 2000, 2007).

Oyěwùmí (1997) argues that dominant interpretations of the world draw upon the primacy the West gives to sight and viewing over other senses and hence the common reference to world views. A perspective shared by Levinas (1987:200) who emphasised ‘l’expérience visuelle à laquelle la civilisation occidentale réduit en fin de compte toute vie spirituelle’.^6^ Alternatively, both Oyěwùmí (1997) and Levinas (1987) suggest that people’s understanding of the world and of their immediate environment may be also driven by other senses such as hearing, smelling, touching and tasting. As a result, Oyěwùmí (1997) coined the term *world senses*. In her own words in the context of the Yoruba of Nigeria to whom she belongs:

[*A*] comparative research framework reveals that one major difference stems from which of the senses is privileged in the apprehension of reality – sight in the West and a multiplicity of senses anchored by hearing in Yoruba land. (…) Consequently, relative to Western societies, there is a stronger need for a broader contextualization in order to make sense of the world. (…) A concentration on vision as the primary mode of comprehending reality promotes what can be seen over that which is not apparent to the eye; it misses the other levels and the nuances of existence. (Oyěwùmí 1997:14)

In disaster studies and disaster risk reduction, the primacy of sight is evident. It is obvious in the importance given by physical sciences to reducing hazards, vulnerability and disasters to numbers, graphs and maps. It is too among social sciences, in all their diversity, as well as within circles of practitioners that promote participatory approaches to understanding disaster. If the former focus primarily on written narratives while the latter emphasises different forms of visuals such as drawing and mapping; in both cases, sight is essential. Conversely, there are multiple perspectives that have emerged from various fields of studies, whether these refer to soundscapes or tactility (Murray Schafer 1993; Oyěwùmí 1997; Smith 1994). Such approaches are now promisingly breaking through the cracks of disaster studies (Sou & Webber 2021).

Therefore, it is crucial to recognise otherness and difference. Otherness and difference are here to be recognised as organic rather than the result of a process of alienation and discrimination from the perspective of a putative centre of power, as per the imperialist project of the West (Bhabha 1994; Said 1978; Spivak 1987). Our recognition of organic otherness and difference is to be framed within multiplicity rather than hierarchy, that is, one or some world views and senses and values cannot be considered superior to others. Indeed, it is a skewed and hierarchical perspective on difference that has indeed underpinned the whole colonial and neocolonial agenda and sustained the hegemony of Western scholarship in disaster studies as well as that of policies and actions geared towards reducing the risk of disaster (Gaillard 2021). As Glissant (1990) once argued:

[*L*]a pensée de l’Autre ne cessera d’être duelle qu’à ce moment où les différences auront été reconnues. La pensée de l’Autre ‘comprend’ dès lors la multiplicité, mais d’une manière mécanique et qui ménage encore les subtiles hiérarchies de l’universel généralisant.^7^ (p. 30)

## Ontological pluralism, nativism and hybridity

A pluralist agenda that recognises organic otherness and difference does not entail that non-Western understandings of what we call disaster are necessarily isolated and purely indigenous or ‘original’, that is, deprived of any foreign influences. Our agenda neither promotes an essentialist nor a nativist perspective of the other. Far from it.

Indeed, the effects of past and contemporary processes of globalisation as well as the current reach of universal and normative disaster risk reduction policies and actions led by international institutions, non-government organisations and national governments are likely to be felt around the world, including in the most remote places and societies. This does not mean that these policies and actions have been partly or fully embraced by local people and organisations, but the performative and repetitive nature of the discourses on disaster and associated *dispositif* for disaster risk reduction provides an undeniable environment for apprehending other understandings of what the said discourses and *dispositif* call disaster (Gaillard 2021).

Furthermore, it is this environment that provides the basis for the field of scholarship wherein this very essay sits, that is, disaster studies. The sole recognition of the existence of such field of scholarship and our desire to reinvent, or rather re-imagine, in Ranger’s (1993) terms, its future challenges the very essence of nativism or, in Gilroy’s (1993) terms, ethnic absolutism. A nativist perspective on disaster or the assumption that there are multiple isolated and purely indigenous interpretations of natural phenomena, harm and hardship, would indeed entail that no dialogue is possible, that is, there is no common ground for mutual understanding across cultures and societies (Appiah 1988).

We rather argue that if there are multiple understandings of what we call disaster, these are likely to be hybrid, that is, that they have been through a process of creolisation or *métissage* through interactions with other cultures. These interactions may have preceded Western colonial contact. They may also reflect a process of resistance to colonial power or solely result from the effects of contemporary globalisation. Yet, in all forms of contact, we argue that different understandings of the world co-exist rather than fuse or blend together (Glissant 1997). As such, they are creole rather than integrated. In Glissant’s (1997) own terms:

[*L*]a créolisation n’est pas une fusion, elle requiert que chaque composante persiste, même alors qu’elle change déjà. L’intégration est un rêve centraliste et autocratique. La diversité joue dans le lieu, court sur les temps, rompt et unit les voix (les langues). Un pays qui se créolise n’est pas un pays qui s’uniformise.^8^ (p. 210)

He adds that:

[*L*]a créolisation est la mise en contact de plusieurs cultures ou au moins de plusieurs éléments de cultures distinctes, dans un endroit du monde, avec pour résultante une donnée nouvelle, totalement imprévisible par rapport à la somme ou à la simple synthèse de ces éléments.^9^ (Glissant 1997:37)

That creolisation, or *métissage* to use Amselle’s concept (1990), is not only a recent result of globalisation nor only the consequence of colonial rule is critical to our argument. As Amselle (1990) argues, it is likely to be inherent to any identity and society and such a process occurred prior to Western colonial contact in the non-Western world. For example, the common use of Sanskrit loanwords to refer to disaster in both Philippine and Indonesian languages, *sakuna* and *bencana,* respectively, reflects the influence of pre-colonial trading interactions with South Asia. As such, it is doubtful that there was any clearly distinct, isolated and unique understanding of the world, including of disaster, prior to colonial contact. Rather, people’s interpretation of their environment and experiences has always been fluid and hybrid, interacting and borrowing from each other (Amselle 1990). This assumption is essential because it allows us to move beyond the existence of distinct and hierarchical categories and classifications, whether it is for identities or societies at large, that we challenge in *The Invention of Disaster*. Our point was indeed that such categories and classifications, inherited from Enlightenment thinking, have supported the imperialist project of the West in promoting hegemonic interpretations of disaster and imposing normative policies for disaster risk reduction, which assume that Western understandings of the world, including of what we call disaster, are superior and universal.

Furthermore, the process of creolisation does neither result from the passive subjection of one form of knowledge to another, of one culture to another, or of one group to another, as per the expectation of the imperialist and colonial project of the West that assumed that its world view, science and praxis are inherently better and universal and hence to be rolled out around the rest of the world as the absolute truth. There is considerable literature from very different perspectives that have shown that hybridity and creolisation rather reflect a process of resistance to the exercise of power, including colonial rule (Bhabha 1994; De Certeau 1980; Prakash 1999; Rafael 1988; Scott 1990; Young 1993). This tactical subversion, in Scott’s (1990) terms, has allowed those who were or are still oppressed to cope and often covertly overturn unequal power relations to their advantage.

This process of hybridisation or creolisation through resistance is not necessarily frontal and violent but always reflect unequal power relations. Bhabha (1985:153) argues that ‘resistance is not necessarily an oppositional act of political intention, nor is it the simple negation or exclusion of the “content” of another culture, as a difference once perceived’. In Bhabha’s (1985, 1994) perspective, hybridity results from the epistemological impossibility of the imperialist project of the West, including to imposing one single understanding of disaster and universal sets of actions for disaster risk reduction, to achieve its goal. This is because this imperialist project is fundamentally grounded in a hierarchical understanding of cultures, where the West holds the absolute truth and the rest of the world is inferior. Therefore, one cannot expect those who are inferior and dominated to fully absorb and mimic Western culture or it would undermine the whole ethos underneath the said imperialist project. It is in this tension that hybridity finds its roots. For Bhabha (1985), both resistance and hybridity are thus:

[*T*]he effect of an ambivalence produced within the rules of recognition of dominating discourses as they articulate the signs of cultural difference and reimplicate them within the deferential relations of colonial power-hierarchy, normalization, marginalization, and so forth. (p. 153)

This revelation led us to suggest in *The Invention of Disaster* that local people in Africa, Latin America and across Asia and the Pacific may be both subjected to Western perspectives on disaster and the ‘recipients’ of standardised disaster risk reduction projects. However, when actually confronted to the challenges of everyday life, including those associated with natural phenomena, the same people might just carry on with their own organic initiatives, as tactical act of subversion and resistance (Scott 1990). Indeed:

[*I*]f the effect of colonial power is seen to be the production of hybridization rather than the noisy command of colonialist authority or the silent repression of native traditions, then an important change of perspective occurs. It reveals the ambivalence at the source of traditional discourses on authority and enables a form of subversion, founded on that uncertainty, that turns the discursive conditions of dominance into the grounds of intervention. (Bhabha 1985:154)

We therefore argue that it is the unique local combination of all forms of interactions between cultures that is at the core of our postcolonial, pluralist agenda for disaster studies. Our contention is that there are many different understandings of what we call disaster around the world not only because there is a myriad of local cultures but also because there are even more diverse forms of interactions that combine and weave together at multiple scales.

For example, in most regions of the Philippines, four different terms are used to refer to what we call disaster in the West: four terms that all reflect different heritages and nuances in harm and hardship. These terms include the very concept of *disaster*, a legacy of the American colonial times and performatively used by the media and the government nowadays. Meanwhile, *kalamidad*, inherited from the earlier Spanish colonial times, is also used, as well as *sakuna*, a Sanskrit loanword that initially meant a bird of omen, and local Austronesian terms, for example, *kasawian* [often used for misfortune] in the Tagalog region of Luzon or *tagku* [misfortune too], in the Kapampangan region, also in the main island of Luzon. These terms cohabit and are all used contingently, depending on the context, in a way that mirrors the hybrid nature of contemporary interpretations of the world, especially of the harm and hardship associated with natural phenomena.

Such pluralist and hybrid approach to understanding disaster henceforth allows us to transcend some of the key legacies of Western imperialism in disaster studies and disaster risk reduction that we uncover in *The Invention of Disaster*. Indeed, it takes our alternative agenda beyond binary interpretations of the world, especially in dealing with so-called marginalised groups and places that stand at the supposed margin of society which centre is the benchmark for safety and flourishment as well as standardised disaster risk reduction. It also enables us to avoid the intellectual aporia of essentialism and nativism by breaking silos and facilitating what Glissant (1990, 2009) called the thoughts of relation and opacity.

## Relation and opacity

Accepting that there may be multiple understandings of what we call disaster that reflect very different world senses and that these understandings of disaster mirror the complex weaving together of multiple historical heritages and contemporary experiences is essential to our agenda. It indeed entails that, according to Glissant (1990:23), ‘toute identité s’étend dans la relation à l’autre’.^10^ As such, our agenda must be both plural and relational.

This plural and relational agenda must be played on a levelled field, which is that relations cannot be hierarchical. Relations across and between cultures thus have to be based on a fair and genuine dialogue. One that recognises organic difference and otherness. One that does not aim for comparison. As Glissant (2009) puts it, it is essential to:

[*R*]econnaître la différence (les différents) comme l’élément premier de la Relation (dans le monde). Le différent, et non pas l’identique, est la particule élémentaire du tissu du vivant, ou de la toile tramée des cultures.^11^ (p. 29)

This dialogue inherently questions our positionality, as researchers and individuals. As Gramsci (1929–1935), once famously suggested:

[*L*]’inizio dell’elaborazione critica è la coscienza di quello che è realmente, cioè un ‘conosci te stesso’ come prodotto del processo storico finora svoltosi che ha lasciato in te stesso un’infinità di tracce accolte senza beneficio d’inventario. Occorre fare inizialmente un tale inventario.^12^ (Q11:XVIII:§12)

It encourages us to reflect upon our relationship to the others, both scholars and people who deal with what we call disaster, recognising that we could share all positions at the same time. It requires humility and empathy as well as compassion and hope (Butler 2010). As Chakrabarty (1995:756) further emphasised, ‘a dialogue can be genuinely open only under one condition: that no party puts itself in a position where it can unilaterally decide the final outcomes of the conversation’.

Furthermore, if one understanding of the world cannot be forced over another or multiple others, then the relation cannot be fully transparent to all parties unfamiliar with the respective world senses and interpretations of what a disaster is. As Young (2004) once reflected:

[*B*]y definition the concept ‘cannot capture the absolutely-other’; and, to the extent that it must invoke a form of generality, of language itself. Any conventional form of understanding must appropriate the other, in an act of violence and reduction. (p. 46)

Translation, as act of violence and reduction, hence becomes the crux of the problem if it is to aim at standardised and transparent definitions of concepts as per the numerous multi-language glossaries available in our field of studies and praxis (Asian Disaster Reduction Center 2002; Toki 1994; United Nations Department of Humanitarian Affairs 1992). In Glissant’s (1990) words:

[*S*]i nous examinons le processus de la ‘compréhension’ des êtres et des idées dans la perspective de la pensée occidentale, nous retrouvons à son principe l’exigence de cette transparence. Pour pouvoir te ‘comprendre’ et donc t’accepter, il me faut ramener ton épaisseur à ce barème idéel qui me fournit motif à comparaisons et peut-être à jugements, Il me faut réduire.^13^ (p. 204)

As a result, our pluralist and relational agenda must accept that our very diverse interpretations of what a disaster is will remain opaque to others. According to Glissant (1990):

[*A*]ccepter les différences, c’est bien sûr bouleverser la hiérarchie du barème. Je ‘comprends’ ta différence, c’est-à-dire que je la mets en rapport, sans hiérarchiser, avec ma norme. (…) Commuer toute réduction. Non pas seulement consentir au droit à la différence mais, plus avant, au droit à l’opacité, qui n’est pas l’enfermement dans une autarcie impénétrable, mais la subsistance dans une singularité non réductible.^14^ (p. 204)

## Ontological pluralism, relativism and legitimation

The foregoing agenda raises obvious epistemological questions, especially with regard to what Latour (2012) calls ‘regimes of truth’. We indeed contend that the dominant normative injunction for scientific validation and legitimation of any truth as absolute, as per the expectations of the modern *épistémè* of the West designed in Europe during the 18th century, is skewed. Lyotard (1979) made that very clear:

[*L*]e scientifique s’interroge sur la validité des énoncés narratifs, et constate qu’ils ne sont jamais soumis à l’argumentation et à la preuve. Il les classe dans une autre mentalité: sauvage, primitive, sous-développée, arriérée, aliénée, faite d’opinions, de coutumes, d’autorité, de préjuges, d’ignorances, d’idéologies. Les récits sont des fables, des mythes, des légendes, bons pour les femmes et les enfants. Dans les meilleurs cas, on essaiera de faire pénétrer la lumière dans cet obscurantisme, de civiliser, d’éduquer, de développer. Cette relation inégale est un effet intrinsèque des règles propres à chaque jeu. On en connait les symptômes. C’est toute l’histoire de l’impérialisme culturel depuis les débuts de l’Occident. Il est important d’en reconnaitre la teneur qui le distingue de tous les autres: il est commandé par l’exigence de légitimation.^15^ (p. 48)

Nonetheless, our agenda does not promote any form of cultural relativism, especially if relativism is about independent and essentialised ontologies and epistemologies, an assumption that we rejected earlier in this article. Our contention that there are multiple hybrid interpretations of what we call disaster rather promotes a dialogue that cultural relativism denies in the first place to the benefits of one single alleged universal and absolute truth (Wiredu 1993). As such, we agree with Latour (2012), that:

[*N*]otre méthode n’implique donc pas d’affirmer que ‘tout est vrai’, que ‘tout se vaut’, que toutes les versions de l’existence, le mal comme le bien, le véritable et le factice, devraient cohabiter sans qu’on se soucie plus de les trier (…). Mais seulement que le tri devra se faire, dorénavant, à armes égales.^16^ (p. 154)

We thus argue that there are multiple truths that must co-exist in a non-hierarchical perspective. This is what Glissant (2009) called non-absolute truths.

The raising concern is hence about the process(es) of legitimation and validation of multiple truths in their very own and unique context rather than against a universal and absolute benchmark. Our current theoretical frameworks and models as well as our standardised methodologies inherited from the Western modern *épistémè* are indeed unable to capture and appreciate diverse understandings of disaster because their alleged universality draws upon a legitimacy that the West has itself founded on a skewed and hierarchical approach to difference. One that is based on the assumption that Western knowledge and science is inherently superior to other understandings of the world (Bhabha 1994; Lyotard 1979; Mignolo 2000, 2007). One that assumes that sight is the primary sense to mobilise to understand and reduce disaster risk.

As a result, our agenda entails that we move our understanding of truth, of what a disaster may be and may not be, and hence how to deal with such challenges, from objectivity to trust. Trust that others’ different understandings of the world are as valid as those inherited from the Enlightenment and the modern *épistémè* of the West carved in Europe’s 18th century. Trust that should replace a ‘worship of “objectivity”’ in Constantino’s (1978:283) terms. It is about ‘faire la “vérité”, sa “vérité”, dans la condition humaine une vérité d’abord croyable à soi-même, sans mystification’^17^ (Eboussi-Boulaga 1977:155).

## The *Tout-Monde* of disaster studies

The combined additions of all different and hybrid others in their multiple unique interactions compose Glissant’s *Tout-Monde*, which is usually translated as *Whole-World* in English but which we prefer to use in French for the limitations of translation exposed in the previous section. The *Tout-Monde* of disaster studies is therefore an archipelago composed of all the different and hybrid interpretations of what we call a disaster around the world; again, if ever the concept makes any sense at all beyond the West. The whole, that is, the archipelago is therefore diverse and relational rather than homogenous and universal. It is complementary rather than hierarchical. In Glissant’s (1997) own words:

[*L*]a trame du monde s’avive de toutes les particularités, quantifiées; de tous les lieux, reconnus. La totalité n’est pas ce qu’on a dit être l’universel. Elle est la quantité finie et réalisée de l’infini détail du réel. Et qui, d’être au détail, n’est pas totalitaire.^18^ (p. 192)

In that sense Glissant’s *Tout-Monde* meets Mignolo’s (2000) and Escobar’s (2018) idea of the Pluriverse, that is:

[*A*] world in which many worlds could co-exist can only be made by the shared work and common goals of those who inhabit, dwell in one of the many worlds co-existing in one world and where differences are not cast in terms of values of plus and minus degree of humanity. (Mignolo 2007:499)

Exploring the *Tout-Monde* of disaster studies will constitute a radical and forward-looking postcolonial agenda; radical because it will challenge many of our scholarly assumptions, popular discourses as well as common-sense policies and practices. It will embrace plurality. As such, it is neither a new hegemonic paradigm nor an alternative universal *épistémè*. This agenda is rather a call for other approaches to emerge and, at the same time, a space wherein these approaches will flourish. It will entail to consider the five key dimensions of Glissant’s own so-called archipelagic agenda, that are, the *thought of trembling*, the *thought of wandering* or *errantry*, the *thought of creolisation*, the *thought of opacity* and, finally, the *thought of relation*.

The *thought of trembling* is about considering that all things related to disaster are fluid in time. Concepts and processes, people and identities, as well as practices and stakeholders are not static. They constantly evolve in interaction with multiple others. Foreign influences continue to weave together with local perspectives under the pressure of globalisation, neoliberalism and neocolonialism. There cannot, therefore, be any standard concepts, definitions, methodologies and even theories available to help use make sense of challenges in people’s everyday life, whether these are called disaster or not. In this perspective, Glissant (2009) speaks of sinuation in opposition to insinuation. The latter would be dictated by normalising assumptions for what we want to find while the former entails that we should be guided by the diversity of local contexts that we navigate and explore driven by curiosity.

This journey is therefore one of wandering or errantry, where nothing is fixed in space nor associated with a particular origin that would dictate its truth. In Glissant’s (2009) own words:

[*P*]ar la pensée de l’errance nous refusons les racines uniques et qui tuent autour d’elles : la pensée de l’errance est celle des enracinements solidaires et des racines en rhizome. Contre les maladies de l’identité racine unique, elle est et reste le conducteur infini de l’identité relation.^19^ (p. 61)

Wandering through cultures and heritages, across places and world senses, neither suggests that we throw all what we know out of the window nor that we abandon all the concepts (including disaster and all its cognates), methodologies and theories, in all their own diversity, that we use today. As Derrida (1967:25) once said ‘nous devons d’autant moins renoncer à ces concepts qui nous sont indispensables pour ébranler aujourd’hui l’héritage dont ils font partie’.^20^ Wandering through multiple, diverse and interrelated perspectives on disaster rather entails that we:

[*C*]ontinue to use them [*i.e. the concepts*], to repeat them, to repeat them subversively, and to displace them from the contexts in which they have been deployed as instruments of oppressive power. (Butler 1995:51)

It is through this critical *trembling* and *wandering* journey that we will be able to fully embrace the *thought of creolisation*. That we will be able to fully capture local indigenous perspectives on disaster but also how they weave with, nuance, and complement foreign influences, whether future, recent or older. Considering hybridity and creolisation in our research, endeavour will thus be a dynamic process, both in time and space. Glissant (2009:64) adds that:

[*C*]’est processus, et non pas fixité. Il y a une alchimie de la créolisation, qui outrecroise les métissages, et quand même elle passe par eux. J’ai ainsi proposé le mot, qui a naturellement (ou par force) été cueilli (accueilli) partout, rejoignant sa réalité.^21^ (p. 64)

This process will further allow us to explore how normative foreign influences, for example, with regard to disaster risk reduction (Gaillard 2021), are embraced or resisted by local people when confronted with challenges in everyday life. As Bhabha (1985) argued, ‘the hybrid object, however, retains the actual semblance of the authoritative symbol but revalues its presence by resiting it’.

Fully understanding the complex, unique and hybrid nature of local perspectives on disaster will be the privilege of local people or those who are grounded in particular places. To others, these local interpretations of disaster will likely remain opaque, which is what underpins Glissant’s (1997) *thought of opacity*:

[*J*]e réclame pour tous le droit à l’opacité, qui n’est pas le renfermement. C’est pour réagir par là contre tant de réductions à la fausse clarté de modèles universels. Il ne m’est pas nécessaire de ‘comprendre’ qui que ce soit, individu, communauté, peuple, de le ‘prendre avec moi’ au prix de l’étouffer, de le perdre ainsi dans une totalité assommante que je gérerais, pour accepter de vivre avec lui, de bâtir avec lui, de risquer avec lui.^22^ (p. 29)

Accepting opacity is not isolating or alienating. It is an act of solidarity and mutual trust. According to Glissant (1990) himself:

[*J*]e puis donc concevoir l’opacité de l’autre pour moi, sans que je lui reproche mon opacité pour lui. Il ne m’est pas nécessaire que je le ‘comprenne’ pour me sentir solidaire de lui, pour bâtir avec lui, pour aimer ce qu’il fait. Il ne m’est pas nécessaire de tenter de devenir l’autre (de devenir autre) ni de le ‘faire’ à mon image.^23^ (p. 207)

We thus need to encourage the use of local languages to the detriment of the violent and reductionist act of translation in a foreign tongue.

Opacity therefore allows rather than obstructs the *thought of relation*. The *thought of relation* makes the *Tout-Monde* of disaster studies. It is at its core:

(…) [*L*]a Relation est ici entendue comme la quantité réalisée de toutes les différences du monde, sans qu’on puisse en excepter une seule. Elle n’est pas d’élévation mais de complétude. Ses propositions seraient alors qu’elle s’élargit jusqu’à quantifier absolument cette totalité des différences, c’est-à-dire qu’elle ne se rehausse ni ne se justifie d’aucune sublimité, mais qu’elle parait et se multiplie en continu et s’achève et se prolonge à même cette totalité absolument.^24^ (Glissant 2009:42)

Appraising these multiple, unique, hybrid and opaque relations between places and people, concepts and world senses, as well as across actions and stakeholders, inherently has to draw on a dialogue among us scholars of all backgrounds but also with and among local people and other actors of disaster studies and disaster risk reduction (Hewitt 1994, 1995). Nonetheless, as Glissant (1997:154) argues, ‘*il n’est pas nec essaire de se renier pour s’ouvrir à l’autre*’^25^ as accepting opacity allows such fair dialogue; one that is based on trust, humility, compassion and hope.

Embracing and taking this agenda forward will surely be challenging, especially considering the current hegemonic nature of Western discourses on what we call disaster (Gaillard 2001). However, we believe that it is not impossible. It will require to raise consciousness, in Fanon’s (1952) terms, among local researchers of all locations that there is no universal truth nor common sense when it comes to disasters and that the discourses on disasters that have been reiterated and normalised through the multiple dimensions of the governmentality of disaster risk reduction are not of higher value but rather an instrument of Western imperialism (Gaillard 2021). It is about:

‘[*A*]voir le courage d’affronter l’inconnu est la condition de l’imagination et que la capacité d’imaginer un monde autre est un élément essentiel de la démarche scientifique’ and that ‘pour avancer, il faut d’abord renoncer à certaines évidences; ces ‘évidences’ procurent le sentiment confortable que procurent toutes les certitudes mais elles nous empêchent de poser des questions, ce qui est sinon la seule, au moins la plus sûre façon de parvenir à des réponses’.^26^ (Delphy 1991:89, 96)

This process requires grounding in our own unique interpretations of the world and in our realisation of the unequal and skewed power relations that currently underpin disaster studies and disaster risk reduction. As we suggested in *The Invention of Disaster*, we believe that there are enough creative and committed scholars in Asia and the Pacific, Africa and Latin America to raise consciousness among their peers, set their own priorities, build upon their local epistemologies and hence drive our agenda forward.

## Footnotes

1 ‘How to be oneself without closing oneself to the other, and how to open oneself to the other without losing oneself?’ Quote translated by Muriel Rosemberg in L’Espace Géographique 45(4) of 2016.

2 We agree with Glissant (1981) that the West should be seen as a project of imperialism rather than a mere region of the world. A project that is historically, rather than geographically constructed (Hall 1992).

3 We do not argue that the nature/hazard versus society/vulnerability interpretation of disaster as well as other binary perspectives in understanding the world are only relevant within Europe and its cultural heritage. Nor do we suggest that there is no universal aspiration in diverse non-Western understandings of the world. Our point is that one single binary interpretation of the world and of what disasters has been imposed, through the project of Western modernity and its imperialist ambition, as the only universal truth to the detriment of multiple other perspectives.

4 We recognise that *other* and *otherness* have multiple understandings and that some of them lead to the harmful process of othering, excluding and marginalising, which is antithetical with our argument. However, we stick to these terms in this essay as per Glissant’s own interpretation that is non-hierarchical, inclusive and empowering.

5 ‘The borderline between the normal and the pathological is imprecise for several individuals considered simultaneously but it is perfectly precise for one and the same individual considered successively. In order to be normative in given conditions, what is normal can become pathological in another situation if it continues identical to itself. It is the individual who is the judge of this transformation because it is he who suffers from it from the very moment he feels inferior to the tasks which the new situation imposes on him’. From the English version (pp. 105–106) translated by Carolyn R. Fawcett and published by Zone Books in 1991.

6 ‘[*T*]he visual experience to which Western civilisation ultimately reduces all spiritual life’. Quote translated by Jill Robbins in L’Esprit Createur 35(3) of 1995.

7 ‘Thought of the Other cannot escape its own dualism until the time when differences become acknowledged. From that point on thought of the Other “comprehends” multiplicity, but mechanically and still taking the subtle hierarchies of a generalizing universal as its basis’. From the English version (p. 17) translated by Betsy Wing and published by the University of Michigan Press in 1997.

8 ‘Creolization is not a fusion, it requires each component to persist, even while it is already changing. Integration is a centralist and autocratic dream. Diversity is at play in the place, runs across different times, breaks and unites voices (languages). A country that creolizes is not a country that becomes uniform’. From the English version (p. 130) translated by Celia Britton and published by Liverpool University Press in 2020.

9 ‘Creolization is the putting into contact of several cultures or at least several elements of distinct cultures, in a particular place in the world, resulting in something new, completely unpredictable in relation to the sum or the simple synthesis of these elements’. From the English version (p. 22) translated by Celia Britton and published by Liverpool University Press in 2020.

10 ‘[*E*]very identity is extended through a relationship with the Other’. From the English version (p. 11) translated by Betsy Wing and published by the University of Michigan Press in 1997.

11 ‘[*T*]o recognise difference (the differences) as the prime component of Relation (in the world). The difference, and not the similarity, is the elementary particle of the human fabric, or of the matrix of cultures’. Our translation.

12 ‘The starting-point of critical elaboration is the consciousness of what one really is, and is ‘knowing thyself’ as a product of the historical process to date, which has deposited in you an infinity of traces, without leaving an inventory. Therefore it is imperative at the outset to compile such an inventory’. From the English version translated by Quintin Hoare and Geoffrey Nowell Smith and published by International Publishers in 1971.

13 ‘[*I*]f we examine the process of “understanding” people and ideas from the perspective of Western thought, we discover that its basis is this requirement for transparency. In order to understand and thus accept you, I have to measure your solidity with the ideal scale providing me with grounds to make comparisons and, perhaps, judgments. I have to reduce’. From the English version (pp. 189–190) translated by Betsy Wing and published by the University of Michigan Press in 1997.

14 ‘[*A*]ccepting differences does, of course, upset the hierarchy of this scale. I understand your difference, or in other words, without creating a hierarchy, I relate it to my norm. (…) Displace all reduction. Agree not merely to the right to difference but, carrying this further, agree also to the right to opacity that is not enclosure within an impenetrable autarchy but subsistence within an irreducible singularity’. From the English version (p. 190) translated by Betsy Wing and published by the University of Michigan Press in 1997.

15 ‘The scientist questions the validity of narrative statements and concludes that they are never subject to argumentation or proof. He classifies them as belonging to a different mentality: savage, primitive, underdeveloped, backward, alienated, composed of opinions, customs, authority, prejudice, ignorance, ideology. Narratives are fables, myths, legends, fit only for women and children. At best, attempts are made to throw some rays of light into this obscurantism, to civilize, educate, develop. This unequal relationship is an intrinsic effect of the rules specific to each game. We all know its symptoms. It is the entire history of cultural imperialism from the dawn of Western civilization. It is important to recognize its special tenor, which sets it apart from all other forms of imperialism: it is governed by the demand for legitimation’. From the English version (p. 27) translated by Geoff Bennington and Brian Massumi and published by University of Minnesota Press in 1984.

16 ‘Our method thus does not imply asserting that “everything is true,” “that everything is equal to everything else,” that all the versions of existence, the bad as well as the good, the factitious along with the true, ought to cohabit without our worrying any longer about sorting them out (…). It implies only that the sorting out will have to take place, from now on, on a level playing field’. From the English version (p. 143) translated by Catherine Porter and published by Harvard University Press in 2013.

17 ‘[*T*]o bring out the truth, one’s own truth, in the human condition a truth first and foremost believable by one’s self, without mystification’. Our translation.

18 ‘The weave of the world is enhanced by all the particularities, quantified; by all the places, recognized. The totality is not what has been called the universal. It is the quantity, finished and realized, of the infinite detail of the real. And which, because it is a matter of detail, is not totalitarian’. From the English version (p. 118) translated by Celia Britton and published by Liverpool University Press in 2020.

19 ‘[*T*]hrough the thought of wandering we reject the single roots; those that kill around them: the thought of wandering is one of solidary rooting and rhizomes. Against the sicknesses of single root identity, it is and remains the infinite thread of relation identity’. Our translation.

20 ‘Since these concepts are indispensable for unsettling the heritage to which they belong, we should be even less prone to renounce them’. From the English version translated (p. 14) by Gayatri Chakravorty Spivak and published by the Johns Hopkins University Press in 1974.

21 ‘[*T*]his is process and not fixity. There is an alchemy of creolisation that goes beyond metissages, although the latter are core to the former. I have hence suggested the word, which has naturally (or by force) been picked (welcomed) everywhere, meeting its reality’. Our translation.

22 ‘I claim for everyone the right to opacity, which is not the same as closing oneself off. It is a means of reacting against all the ways of reducing us to the false clarity of universal models. I do not have to ‘understand’ anyone, individual, community, people – i.e. to ‘take them with me’ at the cost of smothering them, of losing them in a boring totality that I would be in charge of – in order to agree to live with them, to build with them, to take risks with them’. From the English version (pp. 16–17) translated by Celia Britton and published by Liverpool University Press in 2020.

23 ‘I thus am able to conceive of the opacity of the other for me, without reproach for my opacity for him. To feel in solidarity with him or to build with him or to like what he does, it is not necessary for me to grasp him. It is not necessary to try to become the other (to become other) nor to “make” him in my image’. From the English version (p. 193) translated by Betsy Wing and published by the University of Michigan Press in 1997.

24 ‘Relation here entails the combined whole of all differences of the world, with no exception. It is not a matter of elevation but of completeness. Its propositions would then suggest that it expands until fully encompassing this totality of differences, that is, it does not increase, nor does it justify itself by any sublimity, but it appears and multiplies continuously and ends and prolongs in this very totality’. Our translation.

25 ‘It is not necessary to reject oneself in order to open up to the other’. From the English version (p. 95) translated by Celia Britton and published by Liverpool University Press in 2020.

26 ‘Having the courage to confront the unknown is a precondition for imagination, and the capacity to imagine another world is an essential element in scientific progress’ and that ‘to advance, we must first renounce some truths. These ‘truths’ make us feel comfortable, as do all certainties, but they stop us asking questions – and asking questions is the surest, if not the only way of getting answers’. From the English version published in Women’s Studies International Forum 16(1) of 1993.
